# Multiplexed amplicon sequencing reveals the heterogeneous spatial distribution of pyrethroid resistance mutations in *Aedes albopictus* mosquito populations in southern France

**DOI:** 10.1186/s13071-024-06632-8

**Published:** 2024-12-27

**Authors:** Albin Fontaine, Antoine Mignotte, Guillaume Lacour, Agnès Nguyen, Nicolas Gomez, Lionel Chanaud, Grégory L’Ambert, Sébastien Briolant

**Affiliations:** 1 Unité des Virus Émergents (UVE: Aix-Marseille Univ, Università di Corsica, IRD 190, Inserm 1207, IRBA), Marseille, France; 2 Institut de Recherche Biomédicale des Armées (IRBA), Unité de Virologie, Marseille, France; 3 Altopictus, Pérols, France; 4 Microsynth France, 170 avenue Gabriel Péri, 69120 Vaulx-en-Velin, France; 5 Institut de Recherche Biomédicale des Armées (IRBA), Unité de Parasitologie et Entomologie, Marseille, France; 6 Aix Marseille Univ, SSA, AP-HM, RITMES, 13005 Marseille, France; 7 IHU Méditerranée Infection, 13005 Marseille, France; 8 Entente Interdépartementale pour la Démoustication du Littoral Méditerranéen (EID Méditerranée), Montpellier, France

**Keywords:** Knock-down resistance, Pyrethroid resistance, Molecular surveillance, Amplicon sequencing, Pool DNA-sequencing, *Aedes albopictus*, Arbovirus

## Abstract

**Background:**

The risk of mosquito-borne disease transmission is increasing in temperate climates with the colonization and proliferation of the Asian tiger mosquito vector *Aedes albopictus* and the rapid mass transport of passengers returning from tropical regions where viruses are endemic. The prevention of major *Aedes*-borne viruses heavily relies on the use of insecticides for vector control, mainly pyrethroids. In Europe, only deltamethrin is authorized.

**Methods:**

High-throughput molecular assays can provide a cost-effective surrogate to phenotypic insecticide resistance assays when mutations have been previously linked to a resistance phenotype. Here, we screened for the spatial distribution of knockdown resistance (*kdr*) mutations at a large scale using a two-step approach based on multiplexed amplicon sequencing and an unprecedented collection of field-derived mosquitoes from 95 sites in 61 municipalities, alongside a west-to-east transect in the south of France, from June to September 2021.

**Results:**

We identified the presence of the V1016G allele in 14 sites. The V1016G allele was predominantly found in southeast France close to the Italian border, with two additional isolated sites close to Bordeaux and Marmande. All mosquitoes were heterozygous for this mutation and should not be phenotypically resistant to pyrethroid insecticide. Four other mutations were identified in our targeted genomic sequence: I1532T, M1006L, M1586L, M995L. Sequencing a section of maternally inherited mitochondrial genome confirmed that the spread of *Ae. albopictus* in France originated from founders within haplogroup A1.

**Conclusions:**

These findings contribute to the broader understanding of resistance dynamics in Europe and can inform targeted approaches to mitigate the impact of resistance on vector control.

**Supplementary Information:**

The online version contains supplementary material available at 10.1186/s13071-024-06632-8.

## Background

The risk of mosquito-borne diseases transmission is now increasing in temperate climates, fostered by the colonization and proliferation of the Asian tiger mosquito vector *Aedes (Stegomyia) albopictus* and the rapid mass transport of passengers returning from tropical regions where viruses are endemic. The unusually high secondary autochthonous cases of dengue virus (DENV) infections in the south of France in 2022 illustrates this risk and is sounding the alarm. The prevention of major *Aedes*-borne viruses heavily relies on the use of insecticides for vector control. In Europe, deltamethrin (a pyrethroid insecticide) is the only insecticide authorized in space spraying to target flying adult mosquitoes [1–3]. Resistance toward this insecticide has been described in *Ae. albopictus* populations throughout the world, including Europe [3–5], yet limited information is available for France. Their spread can negatively impact the effectiveness of vector control interventions and put in jeopardy our very limited defense line.

Monitoring phenotypic insecticide resistance at a large scale is expensive, time-consuming, and laborious. High-throughput molecular assays can provide a cost-effective surrogate when mutations have been previously linked to a resistance phenotype. In addition, molecular methods can detect resistance alleles before they reach fixation and can thus be used as an early-warning approach [6]. Mutations at two codon positions (V1016 and F1534) in the voltage-sensitive sodium channel (*vssc*) gene were experimentally identified as the main knockdown resistance (*kdr*) mechanism to pyrethroids in *Ae. albopictus* [4, 7]. Here, we report a two-step approach based on multiplexed amplicon sequencing to screen for the spatial distribution of *kdr* mutations at a large scale using an unprecedented collection of field-derived mosquitoes sampled from 95 sites across 61 municipalities, alongside a west-to-east transect in the south of France.

## Methods

### Field-collected mosquitoes

*Aedes albopictus* mosquitoes were collected from the field, either at the egg stage using egg-laying traps or at the adult stage using BG sentinel (BGS, Biogents AG) traps, at 95 sites in 61 municipalities alongside a west-to-east transect in the south of France from June to September 2021. Adult mosquitoes were captured over 1 week, with pressurized carbon dioxide bottles provided as a mosquito attractant, and identified morphologically. Mosquito eggs were collected from 181 ovitraps (with a floating extruded polystyrene square of 25 cm^2^) and were hatched according to a standard protocol [8]. Larvae were fed with shrimp food (JBL NovoPrawn) and reared until the fourth instar larvae in laboratories at 23 ± 2 °C and either natural light cycle or a 16:8 h photoperiod. 2833 larvae were recorded by site and sampling date and transferred into 90% ethanol. All samples were stored at −20 °C until the DNA extraction procedure. Traps were all placed at hospitals (areas frequented by symptomatic patients) or at airport and seaport site facilities (points of entry for vectors and arboviruses) covered by the surveillance system deployed under International Health Regulations [9].

### Mosquito DNA extraction

A two-step approach was implemented to screen for *kdr* alleles in *Ae. albopictus* mosquitoes: (i) an initial screening by sequencing pooled mosquito DNA from each site, followed by (ii) sequencing individual mosquito DNA to determine *kdr* allele prevalence and genotype. A total of 3–80 (mean = 24.5, SD = 15) mosquitoes were selected by site and grouped into 100 different pools. Heads from larvae or adult mosquitoes were dissected under magnifying glasses. Each pool was made up of mosquito heads sampled at the beginning and the end of the sampling period for each site when possible. All mosquitoes from sites in which *kdr* alleles were detected in step i were selected for single-mosquito DNA sequencing, excluding damaged mosquitoes. This second selection also included sites without detection of *kdr* alleles in step i, with a total of 56 sites throughout 50 municipalities. Mosquito heads or bodies were ground in a 96 wells plate using a TissueLyser (Qiagen) for 2 min at 30 oscillation/s. Genomic DNA was then extracted from homogenates using the NucleoSpin 96 Tissue Core Kit (Macherey–Nagel) and stored at  −20 °C until use.

### Amplicon-based sequencing

We devised an amplicon-based approach that captured the three main mutations previously reported to be associated with pyrethroid resistance in *Aedes* spp. mosquitoes: S989P, V1016I/G and F1534C/L/S [10, 11]. Two non-overlapping amplicons of 327 and 500 bp were used to amplify two sections of the *vssc* gene that was mapped on the *Aedes albopictus* isolate FPA chromosome 3 chr3.142 whole genome shotgun sequence (AalbF3 genome assembly, GenBank: JAFDOQ010000349.1). This sequence was identified in the AalbF3 genome assembly on the basis of its genetic homology with the *Ae. aegypti* LOC5567355 *vssc* gene sequence. The first and second amplicon mapped to JAFDOQ010000349.1 reference sequence at positions 1,806,101 to 1,806,578 bp and 1,851,149 to 1,851,765 bp, respectively. Both amplicons covered four exons in the *vssc* gene: exon 19-like, exon 20-like, exon 27-like, and exon 28-like. Both targeted genomic regions were amplified in a single reaction to generate sufficient templates for subsequent high-throughput sequencing. Multiplex polymerase chain reactions (PCRs) were performed with 5 μL of purified DNA in a 20 μL reaction mixture made of 5 μL of Hot START 5× Hot Firepol DNA Polymerase mix (Dutscher, France), 1 μL of forward and reverse primers mix at 10 μM (4 μL for four primers) (Additional file 1: Table S1), and 11 μL of water. The thermal program was: 10 min of polymerase activation at 96 °C followed by 35 cycles of (i) 30 s denaturing at 96 °C, (ii) 30 s annealing at 62 °C, and (iii) 1 min extension at 72 °C, followed by a final incubation step at 72 °C for 7 min to complete synthesis of all PCR products. Illumina Nextera^®^ universal tail sequences were added to the 5′ end of each of these primers to facilitate the library preparation by a two-step PCR approach. Our multiplexing design involves a same barcode inserted in both forward primer sequences on each row of a 96 well plates, so that 10 μL of amplified products could be pooled per column (i.e., eight samples were pooled into a single tube with a final volume of 80 μL). This multiplexing scheme allows an 8× sample reduction with 96 samples from one plate being grouped into 12 different tubes, or one plate row (Additional file 2: Fig. S1).

The individual mosquito *kdr* library was complemented with a ligase-based tiling amplicon sequencing method to amplify a 4438-nucleotide region of the mitochondrial genome in each *Ae. albopictus* mosquito. This method generates overlapping amplicons of ~500 base pairs from two multiplexed PCR reactions with six primer pairs in each reaction (Table S1) to generate sufficient templates for subsequent high-throughput sequencing [12, 13]. The Hot START 5× Hot Firepol DNA Polymerase (Dutscher, France) adds an adenosine nucleotide extension to the 3′ ends of each replicated DNA strand to create an A overhang, which makes the product suitable for ligation with T-tailed DNA adaptors. Eight universal barcoded T-tailed DNA adaptors were made by annealing upper and lower oligonucleotides (Table S1) at 25 M in 1× TE and 3 M NaCl buffer, starting with a 1 min step at 95 °C and a constant temperature reduction of −0.1 °C/sec until 12 °C. Each T-tailed DNA adaptor integrated one of the eight barcodes used in the *kdr* library preparation. T-tailed DNA adaptors (1 μL) were diluted to 1.5 µM in water and added to 5 µL of amplicons diluted to 1/10 in water and 5 µL of 2× Blunt/TA Ligase Master Mix (New England Biolabs, Herts, UK), and incubated for 30 min at 25 °C for ligation. No DNA purification was done prior to the ligation step to reduce library costs. Adaptor-ligated amplicons (10 µL) were mixed with 1 µL of the *kdr* library previously diluted 1/10 in water to obtain a *kdr*/mitochondrion (primer pool 1 and 2) library ratio of two, on the basis of DNA concentration, determined by the Qubit fluorometer and Quant-iT dsDNA Assay kit (Life technologies, Paisley, UK) from a random subset of samples. The same barcodes were used to identify one individual across *kdr* and mitochondrial libraries so that the three libraries could be ultimately merged by sample. Amplicons tailed with Illumina Nextera^®^ universal sequences were then pooled by column into a single tube and purified using a 0.8× magnetic bead (SPRIselect, Beckman Coulter) ratio before performing 15 PCR cycles using Nextera^®^ Index Kit PCR primers, that add the P5 and P7 termini that bind to the flow cell and the dual 8 bp index tags. Indexed samples were pooled and quantified by fluorometric quantification (QuantiFluor^®^ dsDNA System, Promega) and visualized on the QIAxcel Capillary Electrophoresis System (Qiagen). Libraries were sequenced on a MiSeq run (Illumina) using MiSeq v3 chemistry with 300 bp paired-end sequencing.

### Data processing and variant calling

The DDemux program [14] was used for demultiplexing FASTQ files according to the P1 barcodes inserted at the 5′-end of each sequence. After demultiplexing, Trimmomatic v0.33 was used to discard reads shorter than 32 nucleotides, filter out Illumina adaptor sequences, remove leading and trailing low-quality bases, and trim reads when the average quality per base dropped below 15 on a four-base-wide sliding window. Reads were aligned to two sections of the JAFDOQ010000349 whole genome shotgun sequence with bowtie2 v.2.1.018 [15]. The alignment file was converted, sorted, and indexed using Samtools v1.6 and BCFtools v1.8 [16]. Coverage and sequencing depth were assessed using bedtools v2.17.0 [17]. DNA variants were called using Lofreq 2.1.5 [18] for pooled-mosquito sequencing, retaining only variants with a quality score greater than 60, and Bcftools mpileup callers for single-mosquito DNA sequencing, respectively.

### Phylogenetic analyses

Consensus mitochondrial sequences were obtained from aligned BAM files using the SAMtools/BCFtools package and seqtk v1.0-r31. Samples were included in the phylogenetic analysis only if at least 30% of their targeted mitochondrial genome section was covered with a base quality score > 20. A background set of 37 full-length mitochondrial genomes were obtained from GenBank [19, 20] to represent the worldwide diversity of *Ae. albopictus* mitogenome haplogroups. The mitochondrial genome of *Ae. aegypti* (NC_035159) was used as an outgroup in the phylogenetic tree. Consensus sequences were aligned using MUSCLE 5.1 [21] and curated by GBLOCKS software implemented in the SEAVIEW version 5.0.4 interface [22] without stringent selection. The curated alignment represented 3243 nucleotides out of the targeted 4438 nucleotides (73%). It was expanded with eight additional samples harboring *kdr* mutations that have between 20% and 30% of their targeted mitochondrial genome section covered with a base quality score > 20. The best-scoring maximum-likelihood (ML) tree was generated using this curated alignment with 120 bootstrap replicates with PhyML [23]. The GTR nucleotide substitution model was chosen on the basis of the lowest Akaike information criterion (AIC) value using the Smart Model Selection (SMS) in PhyML software [24]. Phylogenetic trees were visualized using the ggtree R package [25].

### Statistics and data visualization

Descriptive statistics and data visualization were performed in the statistical environment R v4.2.2 [26]. Figures were made using the package ggplot2 [27], leaflet [28], wesanderson color palette [29], ggtree [25], and the Tidyverse environment [30].

## Results

### Screening of *kdr* mutations in pooled DNA amplicon sequencing

A total of 547 mosquitoes collected from a west-to-east transect in the south of France from June 2021 to September 2021 at 95 sites in 61 municipalities, either at the egg or adult stage, were grouped by sites into 100 pools. Two non-overlapping genomic DNA fragments covering four exons in the *vssc* gene (exon 19-like, exon 20-like, exon 27-like and exon 28-like, as referred to in the JAFDOQ010000349.1 annotation file) were amplified using eight different 6 bp barcodes incorporated at the 5′ end of the forward primers (Table S1). The combination of barcodes and dual indexing allowed the deep sequencing of 13 super-pools, instead of the original 100. The sequencing generated an average depth of 12,779× for amplicon 1 (327 bp, exon 19-like and exon 20-like) and 3336× for amplicon 2 (500 bp, exon 27-like and exon 28-like), per pool after demultiplexing.

A total of 651 mutations were detected on the target region of the *vssc* gene with allele frequencies ranging from 0.1% to 99.9% (median: 3.7%, first quartile: 0.3%, third quartile: 1.4%) (Additional file 3: Fig. S2) across pools. A total of 445 mutations were located on exons, among which 131 (29%) were synonymous and 314 (71%) non-synonymous. These non-synonymous mutations were located at 304 unique positions and had an overall low allele frequency with a median of 0.33% (first quartile: 0.25%, third quartile: 0.51%) across pools (Additional file 4: Table S2). Of these, 17 (5.4%) had mean allele frequencies > 2% across pools (Additional file 5: Fig. S3). The mutations M1006L and I1532T, detected in 98 and 74 pools, respectively, were the most prevalent.

**Fig. 1:**
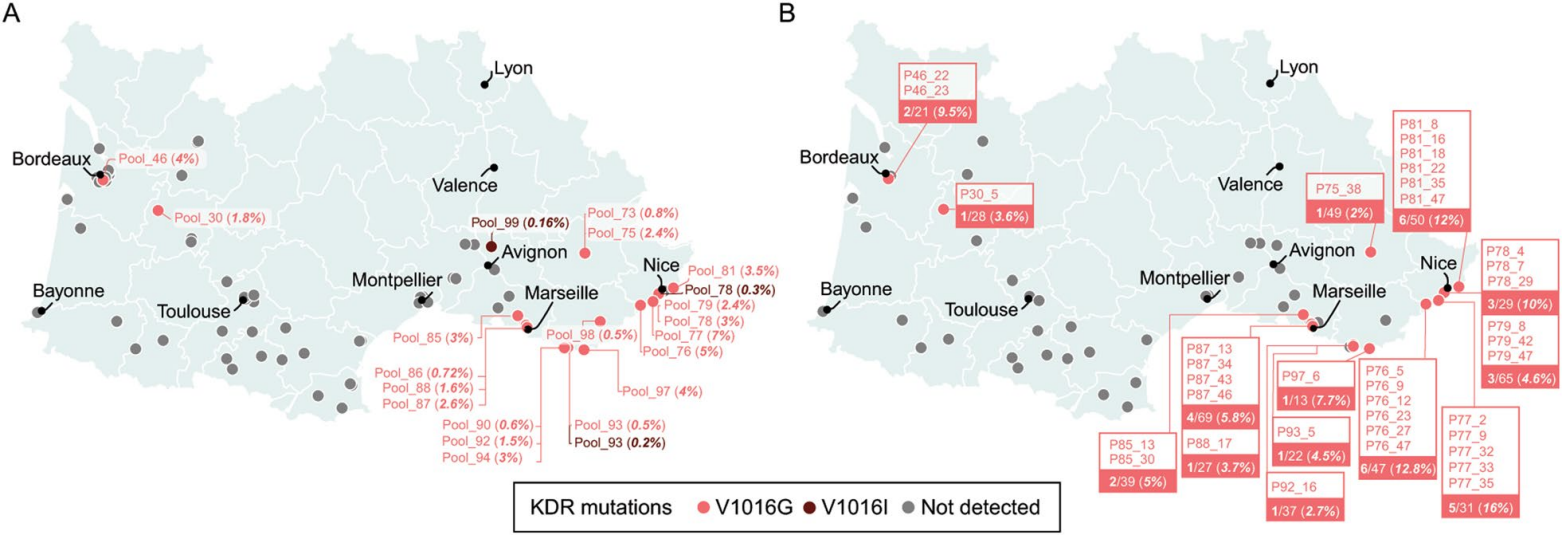
Geographic location of alleles confirmed in knockdown resistance (*kdr*) in *Aedes albopictus* in the south of France. **A** Location and frequencies of *kdr* alleles as revealed by amplicon sequencing based on sequencing of DNA from pooled mosquito heads. Allele frequencies are represented in brackets for each locality. **B** Location and prevalence of *kdr* alleles as revealed by amplicon sequencing on single mosquitoes from each locality. The identity and prevalence of mosquitoes carrying the mutations are represented for each locality. Mosquitoes are identified on the basis of their original pool number and a unique number. Gray points represent localities where no confirmed *kdr* alleles were identified

The *kdr* V1016G and V1016I mutations were detected in 19 and 3 pools, respectively (Fig. 1A). Pools with the V1016I mutation had very low allele frequencies (below 0.625%, which is the theoretical frequency threshold if one heterozygous allele is detected in the biggest pools of *N* = 80). The *kdr* V1016G mutation was preferentially detected in the southeast of France from Marseille to Nice with two exceptions in Bordeaux and Marmande (Fig. 1A).

### Confirmation of *kdr* mutations in single-mosquito DNA amplicon sequencing

Single-mosquito DNA sequencing was implemented to confirm mutations revealed by pool DNA sequencing and to determine their prevalences and genotypes (heterozygote/homozygote). Genetic variations were detected at 135 positions over the target regions of the *vssc* gene. A total of 32 mutations were located on exon-like regions, among which 27 (84%) were synonymous and 5 (16%) non-synonymous: M1006L, M995L, V1016G, I1532T, and M1586L (Additional file 6: Table S3). Importantly, all these mutations were previously identified in the top 20 most frequent mutations in pooled DNA sequencing (Fig. S3). However, some mutations identified in pooled DNA sequencing were not confirmed when sequencing individual mosquito DNA.

The M1006L mutation was the most prevalent (detected in 215 mosquitoes from 45 sites), followed by the I1532T mutation that was detected in 54 mosquitoes from 16 sites. The V1016G mutation was detected in 37 mosquitoes from 14 different sites. This mutation was detected in the same sites as pooled DNA sequencing except for pool_73, pool_86, pool_98, pool_90, and pool_94. Allele frequencies for these pools were mainly < 1% and might be attributed to DNA contamination during the DNA extraction procedure. The V1016G mutation in single-mosquito DNA was not detected in sites where the mutation was not reported by pooled DNA sequencing. All mosquitoes were heterozygous for this mutation. The prevalence of mosquitoes carrying the V1016G mutation ranged from 2% to 16% across sites (Fig. 1B). Single DNA sequencing confirmed the presence of *kdr* V1016G mutation in southeast France, close to the Italian border where it has already been described since 2019 [3, 4], and in a cluster located in Bordeaux and Marmande in the west.

### Geographical dispersion of mosquitoes carrying *kdr* mutations as revealed by their mitochondrial DNA

**Fig. 2:**
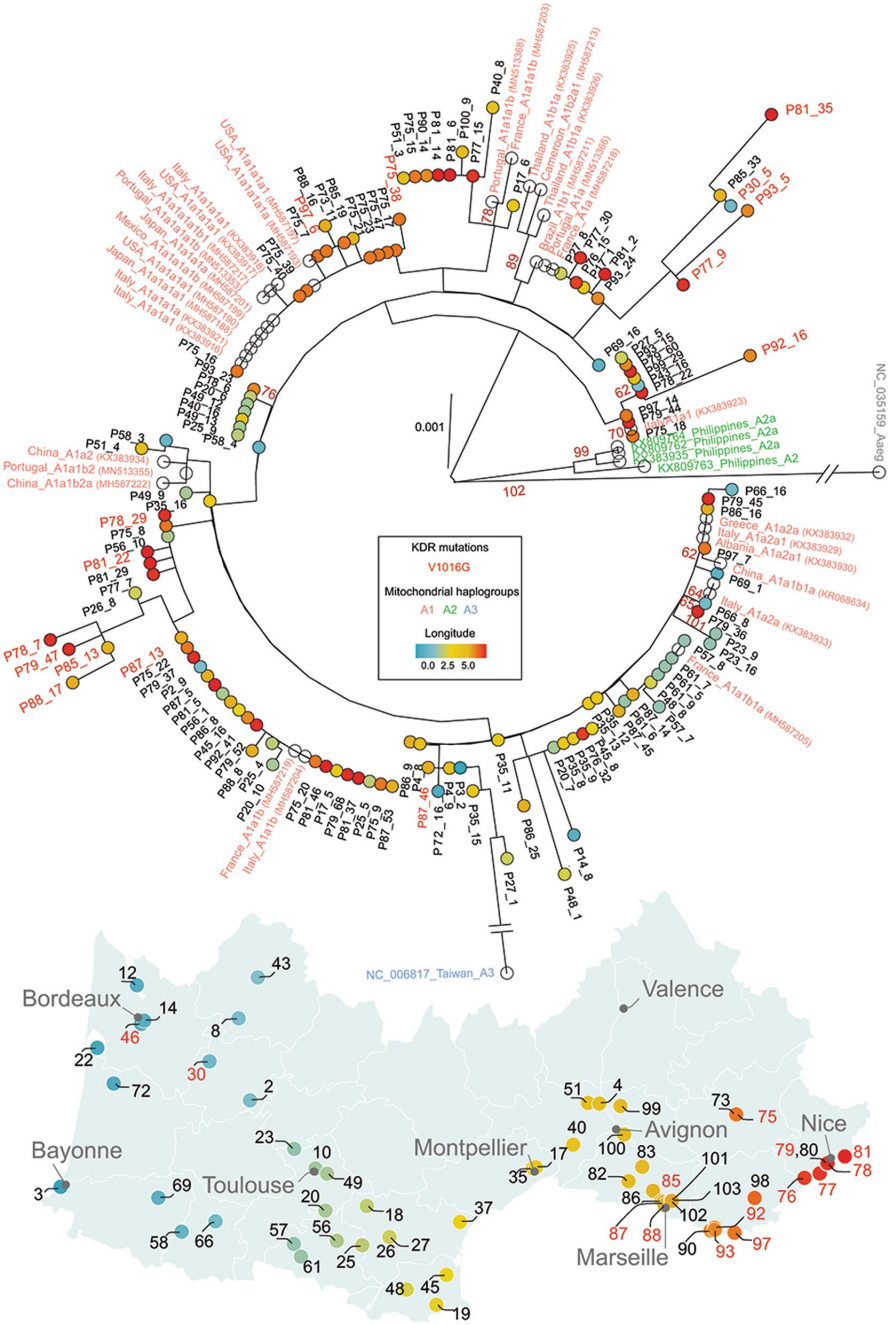
Phylogenetic relationships among a subset of *Aedes albopictus* mosquitoes analyzed in this study were based on a curated alignment of 3243 bp nucleotide region of the mitochondrial genome. The mitochondrial genome of *Ae. aegypti* (NC_035159) was used as an outgroup in the phylogenetic tree. The best-scoring maximum-likelihood (ML) tree was generated with 120 bootstrap replicates. Only bootstrap scores > 60 are represented in dark red. Mosquitoes are identified on the basis of their original pool number and a unique identifier. Pool localities are represented on the map with a color code representing the longitude (west-to-east transect gradient is represented with a blue-to-red color gradient). Localities with at least one mosquito carrying a *kdr* allele are represented in red on both the map and the phylogenetic tree

The amplicon-based library targeting the *vssc* gene was complemented with ligase-based tiling amplicons that amplified a region of the mitochondrial genome in each *Ae. albopictus* mosquito. Genomic regions aligning to the targeted mitochondrial genome had a low read depth (mean ± SD: 5.8× ± 33), as compared with the *vssc* gene. However, a total of 126 samples out of 1167 (11%) could be included in the phylogenetic analysis with background reference sequences that represented the worldwide diversity of *Ae. albopictus* mitogenome haplogroups [19, 20]. The phylogenetic analysis revealed that all collected *Ae. albopictus* mosquitoes originated from founders within haplogroup A1 (Fig. 2). The *kdr* V1016G allele was found in mosquitoes from different maternal lines. One mosquito from the west (P30_5) carrying the *kdr* V1016G mutation had mitochondrial DNA genetically similar to mosquitoes from the east (Fig. 2), suggesting long-range dissemination of the resistance allele through transports.

## Discussion

Pyrethroid insecticides are widely used in agriculture and as indoor/outdoor residual or space spraying for adult mosquito control throughout the world because of their low acute toxicity on mammals and high and fast activity in insects. Mutations in the *vssc* gene were experimentally identified as one of the major knockdown resistance (*kdr*) mechanisms in insects, together with metabolic resistance mainly mediated by P450 monooxygenases [31, 32]. The *kdr* mutations were originally discovered in the model organism *Musca domestica* [33], and mutations found in other insects were named on the basis of the codon position of this house fly reference genome. Several mutations were documented in the *vssc* gene in *Ae. aegypti* (i.e., V410L, S989P, I1011M/V, V1016G/I, I1532T, F1534S/L/C, and D1763Y), but a few of them have been confirmed to be functionally associated with pyrethroid-resistant phenotypes (i.e., V410L, S989P, I1011M, V1016G and F1534C) [10]. Some mutation combinations can engender extreme resistance in *Ae. aegypti*, such as the triple mutant 989P/1016G/1534C haplotype [34]. The *kdr* F1534C mutation was the first to be reported in *Ae. albopictus* in Singapore [7], in 2011, followed by mutations V1016G/I and F1534S/L (alone or in combination) in different parts of the world. The *kdr* V1016G allele was recently found in *Ae. albopictus* populations from Italy, Vietnam [4], and China [35]. In the homozygous state, this mutation was shown to confer a higher level of pyrethroid resistance than the previously known alleles, F1534C and F1534S [4]. The *kdr* V1016G mutation was recently revealed in France in two populations of *Ae. albopictus* from Nice and Perpignan [3].

Here, the spatial distribution of pyrethroid resistance mutations in *Ae. albopictus* populations in southern France was screened in the most exhaustive sampling work to date in France (95 sampling sites across 61 municipalities), using a two-step multiplexed amplicon sequencing approach. We first implemented a sequencing approach using pooled mosquito DNA per site to reduce the overall sequencing costs. This initial step was able to screen for the presence of *kdr* mutations in many sites across a wide study area, faster, and with reduced sample sizes as compared with a single-mosquito DNA screening approach. Several mutations with high allele frequencies and prevalence across sites were detected, including *kdr* V1016G mutations. Importantly, all mutations subsequently confirmed by single-mosquito DNA sequencing were previously identified in pooled DNA sequencing. However, some mutations identified in pooled DNA sequencing were not confirmed when sequencing individual mosquito DNA (e.g., V1016I). This can be partially explained by the allele calling program (LoFreq) applied to pooled DNA sequencing that is more sensitive for distinguishing rare variants than the pipeline applied to single-mosquito DNA sequencing. This can create difficulties in distinguishing rare variants from sequencing errors [36, 37]. The presence of the *kdr* V1016I mutation was not ultimately confirmed by single-mosquito DNA sequencing; this was because of a low allele frequency < 1% in the three pools where it was detected. Pooled DNA sequencing allows the identification of sites with the *kdr* V1016G allele with a perfect sensitivity (100%), albeit not good specificity; with 5 sites out of 19 not confirmed by single DNA sequencing. We suspect that contamination across samples might have occurred during the grinding step prior to the extraction procedure for pooled DNA library preparation. This issue can be easily improved in the future. Furthermore, incorporating unique molecular identifiers (UMIs)—short, random nucleotide sequences added to primers or adaptors during library preparation—can significantly improve the sensitivity of the method. By uniquely tagging each original DNA molecule before amplification, UMIs enable more accurate detection of low-abundance targets, reducing amplification biases and sequencing errors.

By amplifying multiple specific genomic regions of interest using several primers in a single reaction, followed by sequencing, the multiplex amplicon sequencing approach offers a highly efficient solution for analyzing numerous samples and/or targets simultaneously. This minimizes sequencing resources spent on nontarget areas and is effective for detecting low-abundance variants with limited DNA input. However, the method is constrained by PCR amplification errors and a limited number of target regions that can be amplified in a single reaction. In contrast, hybridization capture employs probes to enrich target regions prior to sequencing, making it particularly suited for detecting novel variants outside predefined regions. This approach is ideal for sequencing a wide diversity of genomic regions or entire exomes. However, it is labor-intensive, more expensive than multiplexed PCR-based methods, and requires higher-quality and larger quantities of input DNA. Quantitative capillary sequencing (e.g., Sanger sequencing) is commonly used for sequencing individual or a small number of amplicons. While highly accurate, it is neither cost- nor labor-efficient for sequencing multiple genomic targets across numerous individuals, and is incompatible with pooling strategies that involve mixed DNA samples. An alternative strategy could involve working directly with cDNA spliced from intronic regions to sequence complete *vssc* gene haplotypes with phased genetic variants in single reads using long-read sequencing technologies. Methods that leverage the switch RNA template mechanism (e.g., SISPA [38] or SMART 9N [39]) with specific RT priming could provide a cost-effective solution for sequencing all variants, even in samples with low DNA input.

Our two-step approach can save time and resources, especially when the presence of target mutations is anticipated to be scarce, by excluding samples from sites in which targeted mutations are not detected in a preliminary screening. Efforts and money can then be dedicated in a more efficient way to analyze the prevalence of genotypes using single-mosquito DNA in selected sites. This method can be readily integrated into routine surveillance programs, allowing for the early detection of resistance before the fixation of mutations and the timely implementation of appropriate control measures.

The V1016G allele was predominantly found in southeast France close to the Italian border, with two additional isolated occurrences close to Bordeaux and Marmande. While previous studies have already reported the presence of the *kdr* V1016G allele mutation in Nice and Perpignan, our sampling effort across the south of France did not identify any resistance genes in Perpignan. Importantly, genetic resistance to insecticides can be highly clustered even at a small geographic scale. For example, *vssc* harboring the V1016G allele was not detected from *Ae. albopictus* collected outside of Hanoi in Vietnam, while it was found in the city [4]. In our study, this mutation was found in populations collected from harbor areas in Marseille, but not in those collected more inland from the same city.

The use of pyrethroids is strictly regulated in France when used for curative vector control around human cases of dengue, chikungunya or Zika—imported or autochthonous—to reduce the risk of local arbovirus transmission [3]. Paradoxically, there is no formal ban or monitoring of the use of pyrethroids by pest control companies for nuisance reduction. The use of insecticides by pest control companies or private individuals could maintain a significant selection pressure on local insect populations. Resistance genes carried by *Ae. albopictus* populations in Nouvelle-Aquitaine sites have not been exposed to curative vector control treatments within 150 m since at least 2020. In contrast, resistance genes were not detected in mosquitoes from sites that had been treated six times since 2020. The de novo emergence of mutations is a rare event, and resistance in a population usually arises from the selection of resistance alleles present in a population or from the arrival of individuals with resistance alleles through human transport [6, 40]. Here, we revealed close genetic relationships between mosquitoes collected in the west and east of France, which were carrying the V1016G allele, using a section of the maternally inherited mitochondrial genome. Mitochondrial haplotypes are not suitable to reveal the population structure of *Ae. albopictus* on both small and large scales [41]. Mitochondrial genes often exhibit low resolution owing to limited genetic variation and the potential influence of maternally inherited symbionts, such as Wolbachia. This can obscure fine-scale population differentiation and provide an incomplete understanding of gene flow dynamics. Other genotyping strategies should be used in the future. Altogether, these data suggest that the presence of *kdr* mutations in France originated from fast transportation between distant populations rather than from de novo mutations due to a strong selection pressure.

Although the French Agency for Food, Environmental and Occupational Health Safety (ANSES) issued recommendations on insecticide use and resistance surveillance in the French population in 2020, there is currently no national surveillance program in place [42]. While resistance in vector mosquitoes has been well documented in overseas French territories [32, 43, 44], it remains poorly studied in metropolitan France. The presence of insecticide resistance alleles in *Ae. albopictus* populations from different sites in France highlights the need for a continued monitoring of insecticide susceptibility at a large geographic scale, together with the development of alternative vector control strategies to reduce the selection pressure. All mosquitoes carrying the V1016G mutation in France displayed a heterozygous genotype. Fixation of the *kdr* V1016G allele, and thereby the occurrence of phenotypic insecticide resistance, can arise rapidly in the presence of a strong selection pressure in areas where the allele is detected even at a low prevalence. There is thus a critical need for the implementation of a comprehensive national surveillance program to monitor resistance spatially and temporally in *Ae. albopictus* populations. Such a program would provide valuable insights into the prevalence and spread of resistance, allowing for timely and targeted interventions to maintain the efficacy of vector control measures. This may include reducing treatments, alternating authorized insecticides over space and time, employing complementary methods such as trapping, and innovative control strategies [45, 46] to proactively respond to changes and mitigate the spread of resistance, thereby safeguarding the effectiveness of vector control interventions and protecting public health.

## Conclusions

Our study provides insight into the spatial distribution of pyrethroid resistance mutations in *Ae. albopictus* populations in southern France. We have shown that pooled DNA amplicon sequencing can help to reduce the surveillance costs by detecting the presence of known mutations when they are expected to occur at a low prevalence, prior to screening mosquitoes individually. The use of multiplex amplicon sequencing, with its ability to screen pooled samples and subsequently confirm results by sequencing individual mosquito DNA, is a valuable tool for monitoring the spatial distribution of resistance mutations. The detection of the *kdr* V1016G allele in different French localities highlights the need for continuous monitoring and proactive resistance management strategies. These findings contribute to the broader understanding of resistance dynamics and can inform targeted approaches to mitigate the impact of resistance on vector control efforts.

## Supplementary Information

Additional file 1.

Additional file 2.

Additional file 3.

Additional file 4.

Additional file 5.

Additional file 6.

## Data Availability

Amplicon-based metabarcoding raw sequencing data are accessible under the NCBI BioProject number PRJNA1120364.
