# Supplementary figures and images for: Multiplexed amplicon sequencing reveals the heterogeneous spatial distribution of pyrethroid resistance mutations in Aedes albopictus mosquito populations in southern France
Source: Parasit Vectors. 2024 Dec 27;17:539. doi: 10.1186/s13071-024-06632-8 (PMC11674153; doi:10.1186/s13071-024-06632-8)

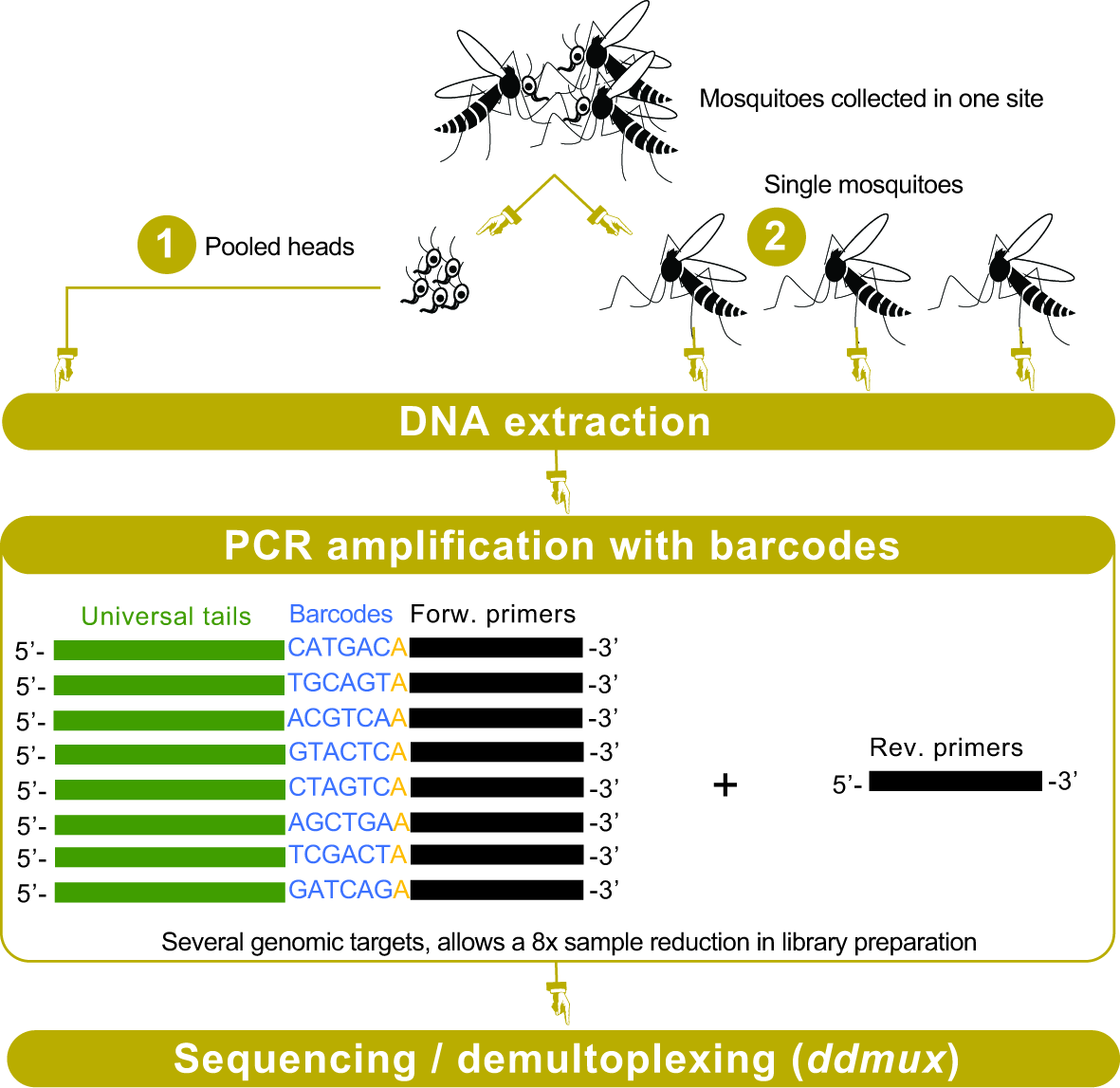

Supplement: Supplementary file 2 — Additional file 2. [file 13071_2024_6632_MOESM2_ESM.tif]

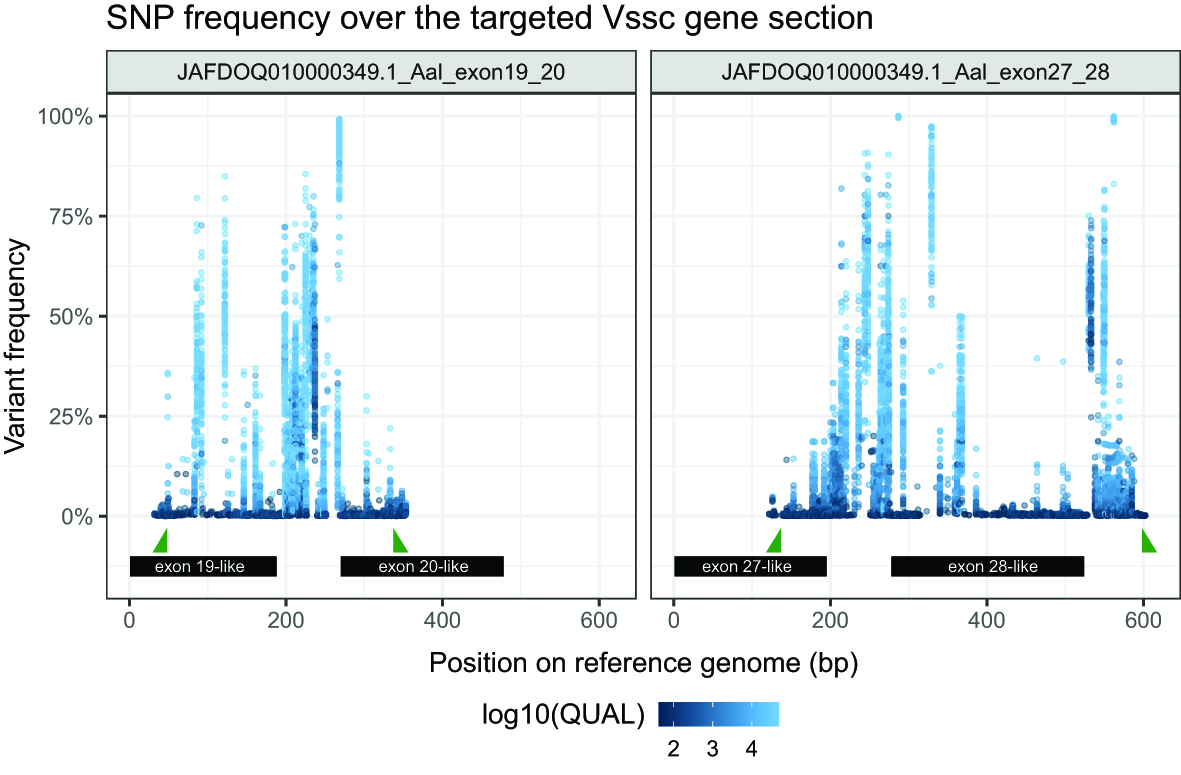

Supplement: Supplementary file 3 — Additional file 3. [file 13071_2024_6632_MOESM3_ESM.tif]

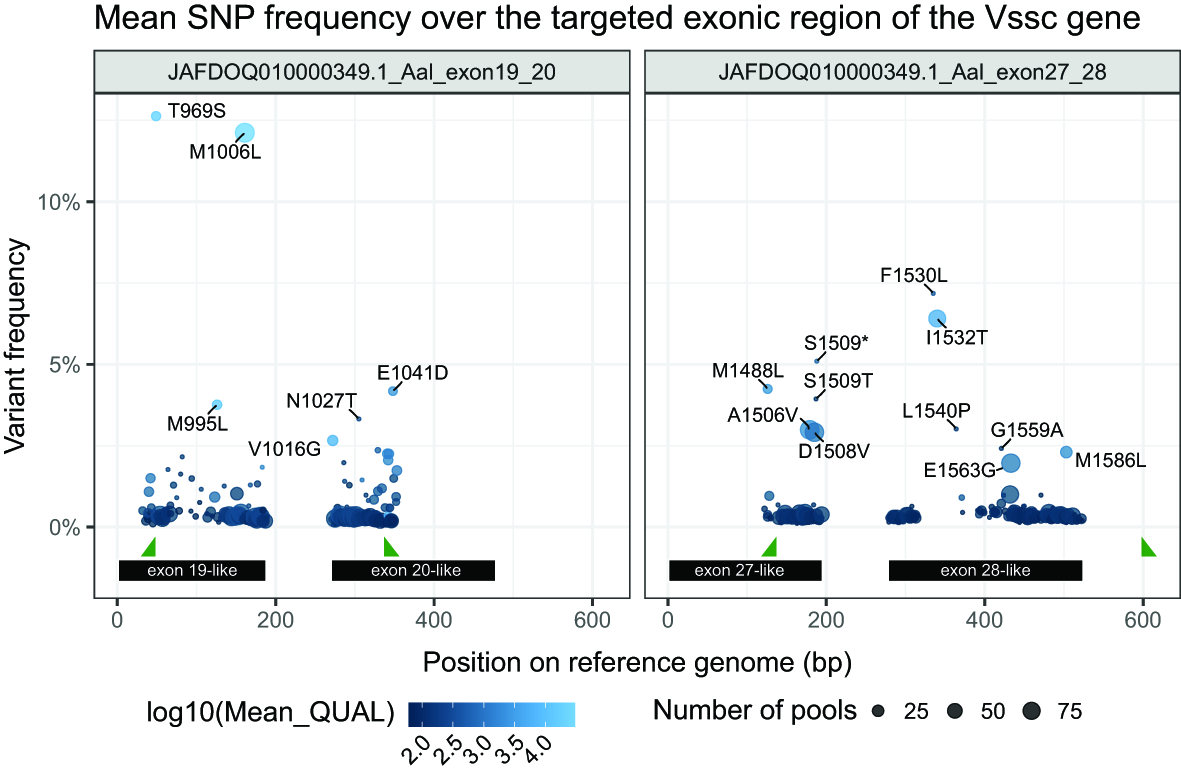

Supplement: Supplementary file 5 — Additional file 5. [file 13071_2024_6632_MOESM5_ESM.tif]
